# Genomic characterization of carbapenem-resistant *Klebsiella oxytoca* complex in China: a multi-center study

**DOI:** 10.3389/fmicb.2023.1153781

**Published:** 2023-07-03

**Authors:** Weimin Wan, Xiaochun Yang, Hua Yu, Min Wang, Wei Jia, Bin Huang, Fen Qu, Bin Shan, Yi-Wei Tang, Liang Chen, Hong Du

**Affiliations:** ^1^Department of Clinical Laboratory, The Second Affiliated Hospital of Soochow University, Suzhou, Jiangsu, China; ^2^Department of Clinical Laboratory, The Affiliated Suzhou Hospital of Nanjing Medical University, Suzhou Municipal Hospital, Gusu School, Nanjing Medical University, Suzhou, Jiangsu, China; ^3^Sichuan Academy of Medical Science and Sichuan Provincial People's Hospital, Chengdu, Sichuan, China; ^4^Center of Medical Laboratory, The General Hospital of Ningxia Medical University, Yinchuan, China; ^5^Department of Laboratory Medicine, The First Affiliated Hospital of Sun Yat-sen University, Guangzhou, Guangdong, China; ^6^Laboratory Medicine Center, Aviation General Hospital, Beijing, China; ^7^Department of Laboratory Medicine, The First Affiliated Hospital of Kunming Medical University, Kunming, Yunnan, China; ^8^Department of Medical Affairs, Danaher Diagnostic Platform/Cepheid (People's Republic of China), New York, NY, United States; ^9^Center for Discovery and Innovation, Hackensack-Meridian Health, Nutley, NJ, United States; ^10^Hackensack Meridian School of Medicine, Seton Hall University, Nutley, NJ, United States

**Keywords:** *Klebsiella oxytoca* complex, resistance, carbapenemase gene, plasmid, MLST

## Abstract

Carbapenem-resistant (CR) *Klebsiella oxytoca* complex can be associated with high mortality, emerging as a new threat to the public health. *K. oxytoca* complex is phylogenetically close to *K. pneumoniae*, one of most common species associated with multidrug resistance in Enterobacterale. The latest research showed that *K. oxytoca* is a complex of six species. Currently, the epidemiological and genomic characteristics of CR *K. oxytoca* complex in China are still unclear. Here, we conducted a multi-center study on 25 CR *K. oxytoca* complex collected from five representative regions in China. These isolates were, respectively, recovered from respiratory tract (12 cases, 48.0%), abdominal cavity (5 cases, 20.0%), blood (4 cases, 16.0%), urine tract (3 cases, 12.0%) and skin or soft tissue (1 cases, 4.0%). Among them, 32.0% (8/25) of patients infected with *K. oxytoca* complex had a poor prognosis. In this study, three *K. oxytoca* complex species were detected, namely *K. michiganensis*, *K. oxytoca* and *K. pasteurii*, among which *K. michiganensis* was the most common. Three carbapenemase genes were identified, including *bla*_NDM-1_ (10, 38.5%), *bla*_KPC-2_ (9, 34.6%) and *bla*_IMP_ (6 *bla*_IMP-4_ and 1 *bla*_IMP-8_; 7, 26.9%). Subsequent multilocus sequence typing identified various sequence types (STs), among which ST43, ST92 and ST145 were relatively common. Different from the clonal dissemination of high-risk carbapenem-resistant *K. pneumoniae* strains, our research revealed a polyclonal dissemination characteristic of CR *K. oxytoca* complex in China. S1-nuclease PFGE and Southern blot experiment showed that carbapenemase genes were encoded in plasmids of different sizes. Two *bla*_NDM_-harboring plasmids were subsequently sequenced, and were characterized to be IncX3 and IncC incompatibility groups, respectively. This is the first multi-center study of CR *K. oxytoca* complex in China, which improved our understanding of the prevalence and antimicrobial resistance characteristics of CR *K. oxytoca* complex in China.

## Introduction

*Klebsiella oxytoca* is an important member of the *Klebsiella* genus within the family Enterobacteriaceae. *K. oxytoca* is commonly found in the environment, including soil, water, and plants, but also can reside in the intestinal tract as a commensal bacterium (Podschun and Ullmann, 1998; Savino et al., 2009). In patients with a compromised immune system, especially those ICU patients and newborns, *K. oxytoca* can lead to diseases such as antibiotic-associated hemorrhagic colitis (AAHC), bloodstream infections, urinary tract infections, pneumonia, skin and soft tissue infections and intra-abdominal infections (Bouchillon et al., 2013; Hoban et al., 2014; Mineau et al., 2018; Ghasemian et al., 2019). Recently, new findings have significantly advanced our knowledge of this important pathogen. The latest research based on genomic taxonomy has shown that *K. oxytoca* is not a single species, but a complex composed of six species, namely *K. oxytoca*, *K. grimontii*, *K. michiganensis*, *K. huaxiensis*, *K. pasteurii*, and *K. spallanzanii* (Yang et al., 2022).

*Klebsiella oxytoca* complex is mainly reported in Asia-Western Pacific, Western Europe and North America, but rarely in Africa and South America (Yang et al., 2022), and the outbreaks usually involve strains with extended-spectrum beta-lactamases and carbapenamases (Decré et al., 2004; Hoenigl et al., 2012; Lowe et al., 2012). In recent years, carbapenem-resistant *K. oxytoca* (CRKO) complex strains were increasingly detected clinically associated with high mortality, emerging as a new threat to the public health (Hoenigl et al., 2012; Wang et al., 2017). In China, the isolation rate of CRKO complex appears to be relatively low. However, infections caused by CRKO complex frequently lead to serious clinical outcomes in hospitalized patients.

Although the clinical importance of CRKO complex is increasingly recognized, molecular epidemiological studies are still extremely limited in part due to the low detection rate. Up to now, large molecular epidemiological research of CRKO complex is still rare in China, despite that China is regarded as a CRE endemic region. In this study, we collected 25 clinical CRKO complex strains from five hospitals in Beijing, Chengdu, Guangzhou, Yinchuan and Suzhou in China, and conducted a multi-center genomic study, in order to understand the status of antimicrobial resistance and the molecular epidemiological characteristics of CRKO complex in China.

## Materials and methods

### Collection and identification of CRKO complex strains

Our multi-center study includes 5 representative cities in China, namely Beijing, Chengdu, Guangzhou, Suzhou and Yinchuan. All CRKO complex isolates from various clinical samples (sputum, blood and urine, etc.) in hospitalized patients with infections were included, and only the first eligible CRKO complex culture episode per patient was included. Preliminary bacterial species identification was carried out using automatic microbiology analyzer in participating hospitals, and CRKO complex was defined as resistant to at least of one carbapenem (imipenem or meropenem), based on the Clinical and Laboratory Standards Institute (CLSI) guideline. The minimal inhibitory concentrations (MICs) of CRKO complex against carbapenems were provided in the Supplementary Table 1. A total of 25 non-repetitive CRKO complex strains from July 2016 to March 2021 were collected. For this study, all isolates were also identified using matrix-assisted laser desorption ionization-time of flight mass spectrometry (MALDI-TOF MS; Bruker Daltonics, Bremen, Germany) to verify their species.

### Antibiotic susceptibility testing

MICs of isolates against commonly used antibiotics were determined by the Phoenix 100 Automated Microbiology System and the results were interpreted according to the Clinical and Laboratory Standards Institute (CLSI) guidelines (CLSI, 2020). A total of 16 antibiotics were tested, including carbapenems (imipenem and meropenem), cephalosporins (cefazoline, ceftriaxone, ceftazidime, cefuroxime, and cefotaxime), β-lactam inhibitor complex (ampicillin-sulbactam, amoxicillin-clavulanic acid and piperacillin-tazobactam), aminoglycosides (gentamicin and amikacin), monocyclic β-lactams (aztreonam), fluoroquinolones (ciprofloxacin and levofloxacin) and sulphamethoxazole/grimethoprin. For strains only producing KPC-2, novel β-lactam/β-lactamase inhibitor ceftazidime-avibactam susceptibility experiment was also carried out by disk diffusion method.

### Detection of carbapenemase genes

Carbapenemase genes (*bla*_KPC_, *bla*_NDM_, *bla*_IMP_, *bla*_VIM_ and *bla*_OXA-48_) were detected with polymerase chain reaction (PCR; Queenan and Bush, 2007; Nordmann et al., 2011) and other resistance genes were further verified by whole-genome sequencing.

### S1-nuclease PFGE and southern blot

In order to study the size of the plasmids harboring resistance genes (*bla*_KPC_, *bla*_NDM_, *bla*_IMP_), S1-nuclease PFGE and Southern blot experiment was performed on 12 selected strains. The genomic DNA was digested with the endonuclease S1 and then electrophoresed under the following conditions: voltage 6 V/cm, electrophoresis conditions of initial switching time of 2.2 s and final switching time of 63.8 s, running time 18 h, temperature 14°C. Plasmid DNA was then transferred from the gel to the nylon membrane by siphoning. The hybridization probes were prepared and labeled using PCR Dig Probe Synthesis kit (Roche), primer sequences were shown in Supplementary Table 2. DIG Wash and Block Buffer Set (Roche) was used for subsequent washing and blocking, and finally developed by chemiluminescence using CDP-Star. *Salmonella* strain H9812 was used as a size marker.

### Whole genome sequencing (WGS) and bioinformatic analysis

WGS was performed on 25 CRKO complex strains. Genomic DNA was extracted using the Axygen DNA Mini Kit (Axygen, America) and sequenced by the Illumina Miseq system (Illumina, CA, USA) using a paired-end library with an average insert size of 350 bp. The quality filtered reads were assembled *de novo* utilizing SPAdes v3.11 (Bankevich et al., 2012). Average nucleotide identity (ANI) analysis was used to define species. To identify the acquired antimicrobial resistance (AMR) genes, all isolate genomes were genotyped for resistance by ResFinder, a Center for Genomics Epidemiology (CGE) bioinformatics tool (Zankari et al., 2012). The MLST of CRKO complex strains were analyzed by PubMLST^1^ (Jolley et al., 2018).

### The heatmap of genotypic profile and phylogenetic tree of CRKO complex

The phylogenetic relationship of all CRKO complex isolates was conducted with MUMer 3.0, ClonalFrameML and MEGA7.0 as described previously (Liang et al., 2019). The heatmap of genotypic profile and phylogenetic tree were drawn and improved on iTOL (doi: 10.1093/nar/gkab301) and Inkscape v1.0^2^ (Letunic and Bork, 2021). Annotation of resistance genes was carried out using the online database Resfinder (Zankari et al., 2012).

### Plasmid sequencing and bioinformatic analysis

To further detail the characteristics of *bla*_NDM_-harboring plasmids, plasmid conjugation experiments were performed using the mixed broth culture method as previously described (Chen et al., 2014). Transconjugants were identified by detecting carbapenemase genes using PCR. Two representative plasmids isolated from the transconjugants were sequenced by next generation sequencing (NGS). Plasmid DNA was extracted from zygotes using Qiagen Plasmid Midi Kit (Qiagen, Valencia, CA, United States). The sequencing results were latter assembled *de novo* utilizing SPAdes v3.11 (Bankevich et al., 2012). Further assembly was obtained by comparing contigs to reference plasmid sequences (> 99% identical), with additional exanimating of overlapped paired ends and gap closure by PCR.

The plasmid replicon types were analyzed using the PlasmidFinder^3^ (Carattoli et al., 2014). Open reading frames (ORFs) and pseudogenes of assembled plasmid sequences were predicted using *RAST* 2.0 (Brettin et al., 2015) combined with *BLASTP/BLASTN* searches (Boratyn et al., 2013). The annotation of resistance genes, plasmid replicon types, mobile elements and other features were carried out using the online databases including *CARD* (Jia et al., 2017), *ResFinder* (Zankari et al., 2012), *PlasmidFinder* (Carattoli et al., 2014), *ISfinder* (Siguier et al., 2006), *INTEGRALL* (Moura et al., 2009), and *Tn Number Registry* (Tansirichaiya et al., 2019). Gene organization diagrams were drawn in *Inkscape 0.48.1* software^4^ (Jolley and Maiden, 2010).

### Nucleotide sequence accession numbers

The sequences of the 25 CRKO complex strains were submitted to GenBank under BioProject PRJNA925298. The sequences of two mapped plasmids were submitted to GenBank under accession number OQ338157-OQ338158.

## Results

### Characteristics of bacterial isolates

We collected a total of 25 non-repetitive CRKO complex strains from five hospitals in five China cities (Beijing, Chengdu, Guangzhou, Suzhou, and Yinchuan). The clinical and molecular characteristics were shown in Table 1. The sequencing analysis results showed that 16 strains were *K. michiganensis*, 5 strains were *K. oxytoca*, and 4 strains were *K. pasteurii*. These isolates were recovered from respiratory tract (12 cases, 48.0%), abdominal cavity (5 cases, 20.0%), blood (4 cases, 16.0%) and urine tract (3 cases, 12.0%), and one case was from skin or soft tissue. 32.0% (8/25) of patients had a poor prognosis.

**Table 1:** Clinical and molecular characteristics of carbapenem-resistant (CR) *Klebsiella oxytoca* complex.

Isolate	City	Species	Gender	Age (years)	Ward	Specimen	Outcome	Carbapenemase	ST
*K. ox*1962	Chengdu	*Klebsiella pasteurii*	F	88	Geriatrics	Urinary tract	Improved	NDM-1	193
*K. ox*2641	Chengdu	*Klebsiella oxytoca*	M	89	Respiratory	Respiratory tract	Death	NDM-1	282
*K. ox*2691	Chengdu	*K. michiganensis*	M	67	ICU	Respiratory tract	Improved	KPC-2	43
*K. ox*4379	Chengdu	*K. michiganensis*	M	88	ICU	Respiratory tract	Improved	KPC-2	202
*K. ox*4543	Chengdu	*K. michiganensis*	M	71	Gastroenterology	Abdominal cavity	Improved	NDM-1	135
*K. ox*6089	Chengdu	*Klebsiella pasteurii*	F	14	Pediatrics	Blood	Improved	-	270
*K. ox*6177	Chengdu	*K. michiganensis*	M	17	Hematology Department	Blood	Death	KPC-2	43
*K. ox*7419	Chengdu	*Klebsiella pasteurii*	F	87	ICU	Blood	Worsened	KPC-2	N1
*K. ox*2871	Guangzhou	*K. michiganensis*	M	65	Pancreaticobiliary surgery	Abdominal cavity	Improved	NDM-1	232
*K. ox*5230	Ningxia	*Klebsiella oxytoca*	M	70	Rehabilitation Division	Urinary tract	Improved	NDM-1	145
*K. ox*1688	Ningxia	*K. michiganensis*	M	8 m	PICU	Respiratory tract	Worsened	NDM-1	43
*K. ox*1344	Suzhou	*K. michiganensis*	M	72	ICU	Blood	Improved	IMP-4	135
*K. ox*1352	Suzhou	*K. michiganensis*	F	79	Oncology Department	Respiratory tract	Worsened	NDM-1	180
*K. ox*2349	Suzhou	*Klebsiella pasteurii*	M	60	Radiotherapy	Respiratory tract	Improved	IMP-4, NDM-1	N2
*K. ox*2708	Suzhou	*Klebsiella oxytoca*	M	41	Neurosurgery	Urinary tract	Improved	IMP-8, KPC-2	2
*K. ox*3097	Suzhou	*K. michiganensis*	M	74	ICU	Respiratory tract	Improved	IMP-4	92
*K. ox*3867	Suzhou	*K. michiganensis*	M	69	General surgery	Abdominal cavity	Improved	NDM-1	85
*K. ox*3873	Suzhou	*K. michiganensis*	M	77	Cardiovascular	Respiratory tract	Worsened	IMP-4	29
*K. ox*4353	Suzhou	*Klebsiella oxytoca*	M	62	Neurosurgery	Respiratory tract	Worsened	KPC-2, NDM-1	145
*K. ox*6915	Suzhou	*K. michiganensis*	F	78	Plastic Surgery	Skin/soft tissue	Improved	IMP-4	92
*K. ox*7525	Suzhou	*K. michiganensis*	M	57	General surgery	Abdominal cavity	Improved	-	91
*K. ox*7566	Suzhou	*Klebsiella michiganensis*	M	40	Hematology Department	Respiratory tract	Worsened	IMP-4	92
*K. ox*7768	Suzhou	*Klebsiella michiganensis*	F	57	Neurosurgery	Respiratory tract	Improved	KPC-2	43
*K. ox*8405	Suzhou	*Klebsiella michiganensis*	F	53	Neurosurgery	Respiratory tract	Improved	KPC-2	108
*K. ox*8859	Suzhou	*Klebsiella oxytoca*	M	77	Outpatient	Abdominal cavity	Unknown	KPC-2	145

*K. ox*, *Klebsiella oxytoca* complex; ICU, intensive care unit; M, male; F, female; m, month.

### Antimicrobial susceptibility tests

The antimicrobial susceptibility of three *K. oxytoca* complex was similar but slightly different (Table 2). All three complex were highly resistant to cephalosporins, penicillin inhibitor complexes and carbapenems, and the susceptibility to the monocyclic β-lactam aztreonam was also low (only 25% of *K. michiganensis* strains were sensitive). Aminoglycosides in general have a high sensitivity, especially amikacin, but in *K. oxytoca*, both aminoglycosides and fluoroquinolones were less sensitive than those in other two complex isolates. For sulphamethoxazole/grimethoprin, only part of strains in *K. michiganensis* were susceptible. Overall, *K. oxytoca* appears to be resistant to more antibiotics than *K. michiganensis* and *K. pasteurii*. However, due to the limited number of *K. pasteurii* and *K. oxytoca* strains in our collection, the drug resistance profile should to be confirmed by further studies.

**Table 2:** Antimicrobial susceptibility of *Klebsiella oxytoca* complex against different antimicrobial agents.

Antibiotics	*Klebsiella michiganensis* (*n* = 16) *n*(%)			*Klebsiella oxytoca* (*n* = 5) *n*(%)			*Klebsiella pasteurii* (*n* = 4) *n*(%)		
	R	I	S	R	I	S	R	I	S
Imipenem	15 (94)	1 (6)	0 (0)	5 (100)	0 (0)	0 (0)	3 (75)	0 (0)	1 (25)
Meropenem	15 (94)	1 (6)	0 (0)	4 (80)	1 (20)	0 (0)	4 (100)	0 (0)	0 (0)
Ampicillin_Sulbactam	16 (100)	0 (0)	0 (0)	5 (100)	0 (0)	0 (0)	4 (100)	0 (0)	0 (0)
Amoxicillin_clavulanic acid	16 (100)	0 (0)	0 (0)	5 (100)	0 (0)	0 (0)	3 (75)	1 (25)	0 (0)
Piperacillin_tazobactam	12 (76)	2 (12)	2 (12)	5 (100)	0 (0)	0 (0)	4 (100)	0 (0)	0 (0)
Cefazolin	16 (100)	0 (0)	0 (0)	5 (100)	0 (0)	0 (0)	4 (100)	0 (0)	0 (0)
Ceftriaxone	14 (88)	1 (6)	1 (6)	5 (100)	0 (0)	0 (0)	4 (100)	0 (0)	0 (0)
Ceftazidime	13 (81)	0 (0)	3 (19)	4 (80)	1 (20)	0 (0)	3 (75)	1 (25)	0 (0)
Cefuroxime	15 (94)	0 (0)	1 (6)	5 (100)	0 (0)	0 (0)	4 (100)	0 (0)	0 (0)
Cefotaxime	15 (94)	0 (0)	1 (6)	5 (100)	0 (0)	0 (0)	4 (100)	0 (0)	0 (0)
Aztreonam	10 (63)	2 (12)	4 (25)	5 (100)	0 (0)	0 (0)	4 (100)	0 (0)	0 (0)
Amikacin	1 (6)	0 (0)	15 (94)	2 (40)	0 (0)	3 (60)	0 (0)	0 (0)	4 (100)
Gentamicin	8 (50)	0 (0)	8(50)	4 (80)	0 (0)	1 (20)	1 (25)	1 (25)	2 (50)
Ciprofloxacin	10 (62)	3 (19)	3 (19)	5 (100)	0 (0)	0 (0)	1 (25)	0 (0)	3 (75)
Levofloxacin	6 (38)	2 (12)	8 (50)	5 (100)	0 (0)	0 (0)	1 (25)	0 (0)	3 (75)
Sulphamethoxazole/grimethoprin	7 (44)	0 (0)	9 (56)	5 (100)	0 (0)	0 (0)	4 (100)	0 (0)	0 (0)

S, susceptible; I, intermediate; R, resistant. *n*(%), *n* = number of isolates; % = percentage.

### Detection of Carbapenemase genes

Carbapenemase genes were further tested in all isolates. A total of 23 out of the 25 (92.0%) strains were found to carry carbapenemase genes, including *bla*_NDM-1_ (10 strains, 38.5%), *bla*_KPC-2_ (9 strains, 34.6%) and *bla*_IMP_ (6 *bla*_IMP-4_ and 1 *bla*_IMP-8_; 7 strains, 26.9%), among which three strains simultaneously contained two carbapenemase genes. Interestingly, strains harboring *bla*_IMP_ were all isolated from Suzhou, accounting for 50% (7/14) of the total number of Suzhou isolates. *bla*_VIM_ and *bla*_OXA-48_ were not detected in this study (Table 1). Notably, carbapenemase genes (*bla*_KPC-2_, *bla*_NDM-1_, and *bla*_IMP_) had significantly different distribution in the three *K. oxytoca* complex (Table 3). *K. michiganensis* had the highest detection rate of *bla*_IMP-4_, while carbapenemase genes in *K. oxytoca* were mainly *bla*_KPC-2_ and *bla*_NDM-1_, and only one strain carried *bla*_IMP-8_.

**Table 3:** Distribution of STs and carbapenemase of CR *Klebsiella oxytoca* complex.

Species	STs (n)	Carbapenemase gene n(%)		
		*bla*_KPC_	*bla*_NDM_	*bla*_IMP_
*Klebsiella michiganensis*	ST43 (4)	3	1	0
	ST92 (3)	0	0	3
	ST135 (2)	0	1	1
	Other STs (7)	2	3	1
	Total (16)	5 (31)	5(31)	5 (31)
*Klebsiella oxytoca*	ST145 (3)	2	2	0
	Other STs (2)	1	1	1
	Total (5)	3 (60)	3 (60)	1 (20)
*Klebsiella pasteurii*	Total (4)	1(25)	2 (50)	1 (25)

### Other resistance genes of the isolates

**Figure 1:**
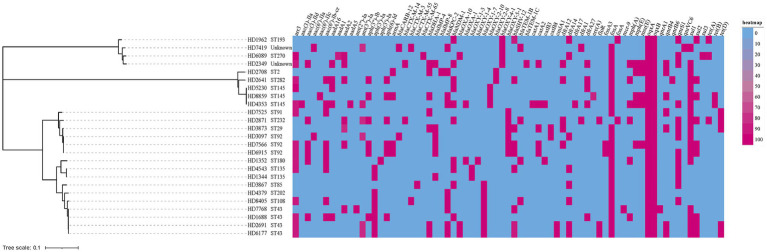
A heatmap of the genotypic profile and phylogenetic tree of all 25 carbapenem-resistant *Klebsiella oxytoca* (CRKO) complex. The cladogram on the left were phylogenetic tree indicating the cluster of isolates by STs. Bar corresponds to the scale of sequence divergence. The genotypic profile was represented as a gene present (red) or absent (blue). The resistance genes were indicated on the column header.

As shown in Figure 1, In addition to the previously confirmed carbapenemase resistance genes by PCR, ESBL-encoding genes (*bla*_TEM_, *bla*_SHV_, and *bla*_CTX − M_) were also identified in *K. oxytoca* complex isolates. And the isolates were confirmed to harbor other resistance genes which mediate resistance to rifampicin (*arr3*), aminoglycoside (*aac(3)-IIa*, *aac(3)-IId*, *aac(6′)-IIa*, *aac(6′)-IIc*, *aac(6′)-Ib-cr, aadA,* etc), phenicols (*catA*, *catB*, *cmlA1*, *floR*), fosfomycin (*fosA*), trimethoprim (*dfrA*), macrolides (*mph(A)*, *mph(E)*, *msr(E)*), fluoroquinolones (*qnrA1*, *qnrB4*, *qnrB6*, *qnrS1*, *qnrVC6*), sulfonamides (*sul1*, *sul2* and *sul3*), and tetracycline (*tet(A) tet(B)* and *tet(D)*). All strains contained efflux pump genes *oqxA* and *oqxB*, and *K.ox*7768 also carried colistin resistance gene *mcr-9*.

### Multilocus sequence typing (MLST)

The results of multilocus sequence typing (MLST) analysis showed that 25 CRKO complex belonged to more than 15 different STs, of which 2 strains belonged to two new STs, named N1 and N2 herein (Figure 1). Except for ST43, ST92 and ST145, which were slightly common (*n* ≥ 3), other STs were mostly detected in a single strain. Results in Table 3 showed that ST43 and ST92 were the main STs of *K. michiganensis*, while ST145 was only detected in *K. oxytoca*.

### Plasmid S1-PFGE and southern blot

Twelve carbapenemase-producing *K. oxytoca* complex isolates were selected for S1-PFGE and Southern blotting to characterize the plasmid sizes of carbapenemase genes. Results showed that plasmids harboring *bla*_NDM_ ranged from 54.7 to 244.4 kb, with sizes of ~55 kb and ~ 150 kb more common. Plasmids harboring *bla*_IMP_ were ~ 40 kb and ~ 173 kb, and plasmids carrying *bla*_KPC_ were 78.2 ~ 104.5 kb (Supplementary Figures 1–3).

### Sequencing of *bla*_NDM_-harboring plasmids

**Figure 2:**
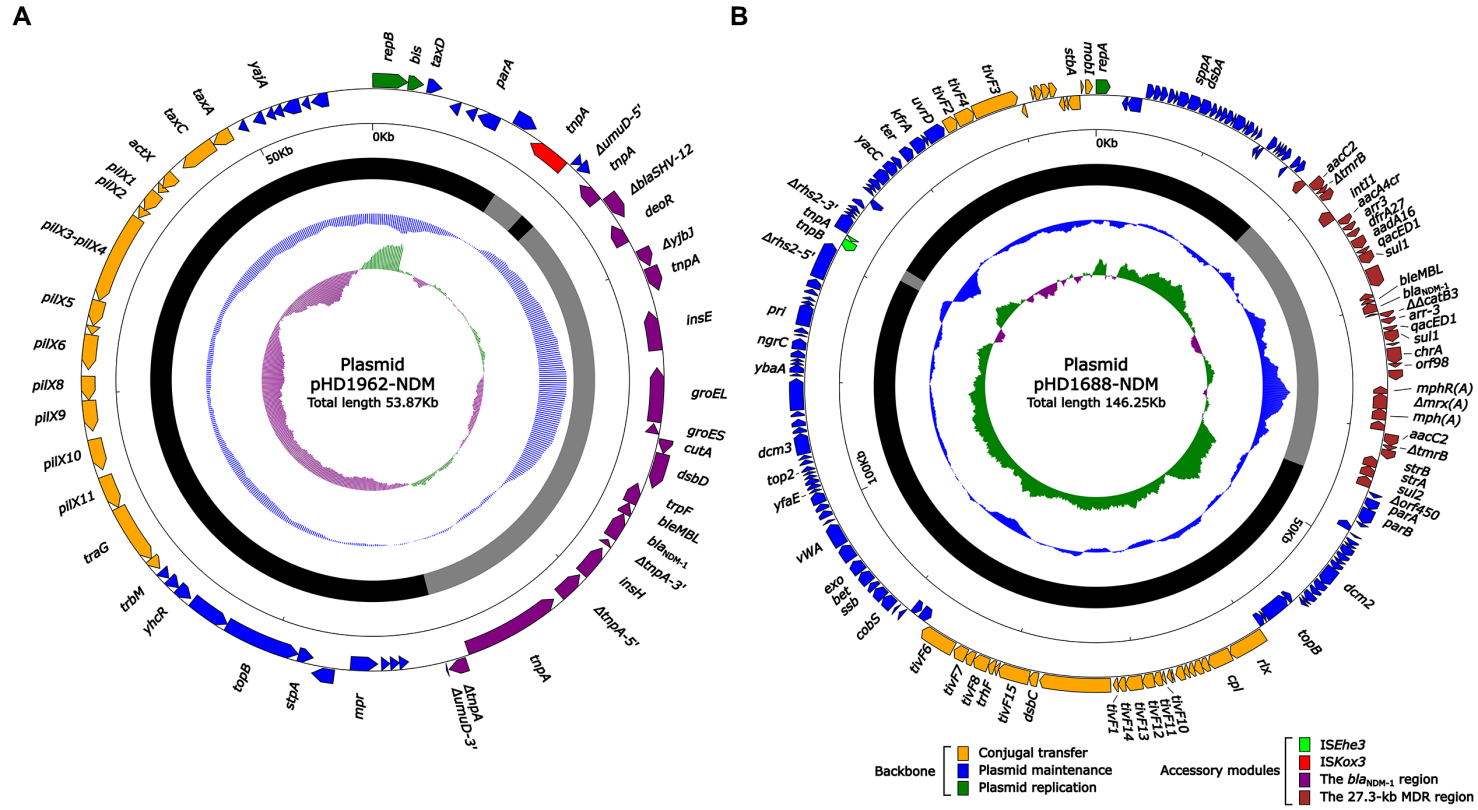
Schematic diagrams of plasmid pHD1962-NDM **(A)** and pHD1688-NDM **(B)**. Genes of different functions are denoted by arrows and presented in various colors. The circles show (from outside to inside): predicted coding sequences, scale in 10 kb, backbone (black) and accessory module (gray) regions, GC content and GC skew [(G–C)/(G + C)].

**Figure 3:**
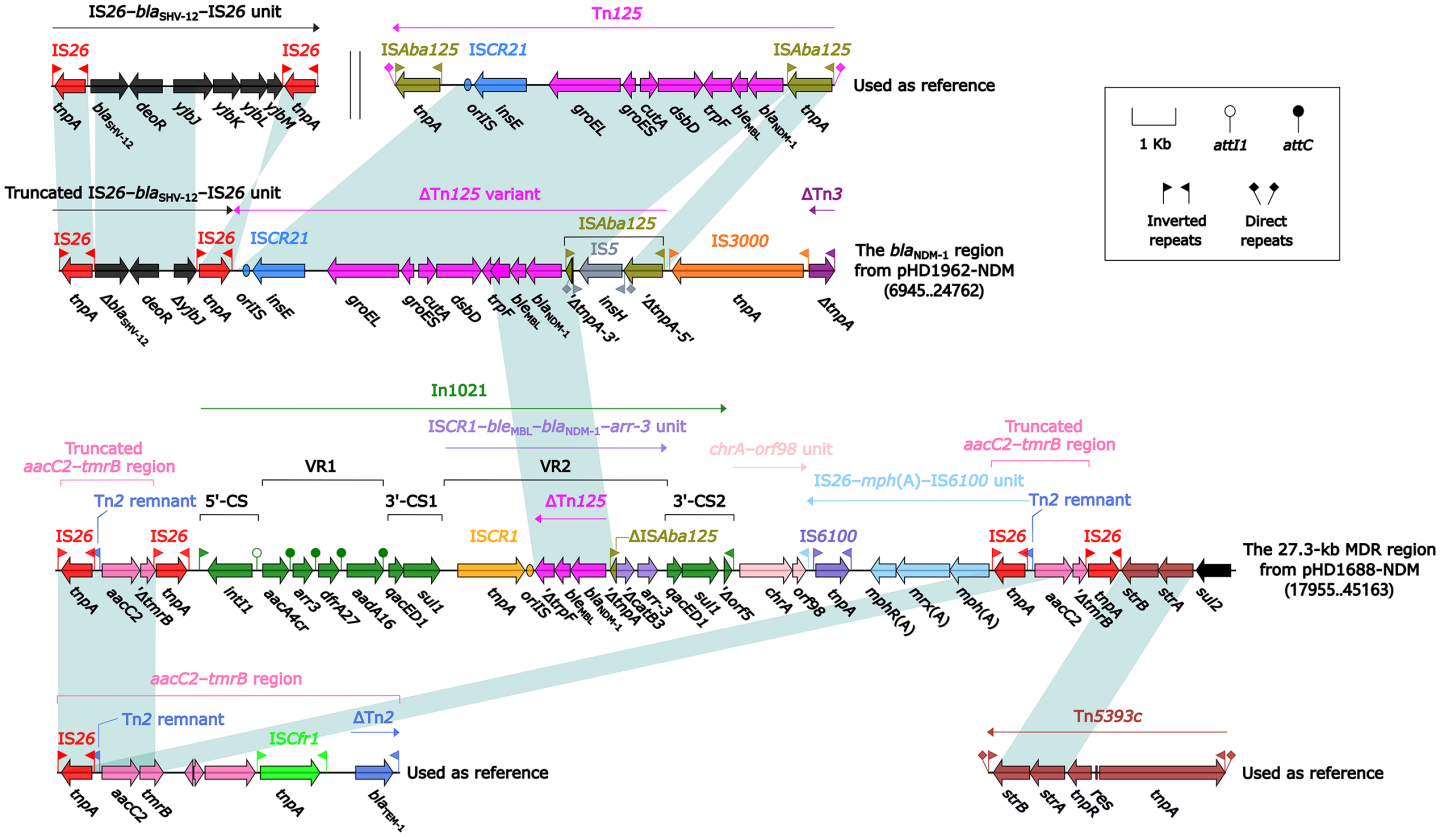
Linear comparison of the *bla*_NDM-1_ region, the 27.3-kb MDR region, and related mobile elements. Genes are denoted by arrows. Genes, mobile elements and other features are colored based on their functional classification. Shading denotes regions of homology (nucleotide identity ≥ 95%). Numbers in brackets indicate nucleotide positions within the plasmid pHD1962-NDM and pHD1688-NDM, respectively. The accession number of IS*26*–*bla*_SHV-12_–IS*26* unit, Tn*125*, *aacC2*–*tmrB* region and Tn*5393c* used as reference are CP024828, JN872328, JX101693 and AF262622, respectively.

S1-PFGE and Southern blotting experiments showed that ~55 and ~ 150 kb were two plasmid sizes with relatively high frequency. We successfully obtained the transconjugants by conjugation experiments and sent two *bla*_NDM_-harboring plasmids (pHD1962-NDM, ~55 kb and pHD1688-NDM, ~150 kb) for further sequencing. Results showed that pHD1962-NDM was encoded by an IncX3 plasmid of 53.87 kb. Except for *bla*_NDM-1_, it also carried another β-lactamase gene *bla*_SHV-12_ (Figure 2A). The pHD1962-NDM carries an AMR region, which comprised of IS*26*–*bla*_SHV-12_–IS*26* unit, truncated Tn*125* variant, IS*3000* and truncated Tn*3*. The *bla*_NDM-1_ is located on a truncated Tn*125* variant (Figure 3). Compared with the reference Tn*125* (Genbank accession number JN872328), the IS*Aba125* downstream IS*CR21* was lost in this truncated Tn*125* variant, while an IS*5* was inserted in the IS*Aba125* upstream of *bla*_NDM-1_. pHD1688-NDM was encoded by an IncC plasmid of 146.25 kb, in which *bla*_NDM-1_ and several other resistance genes were co-located in the 27.3 kb MDR region (Figure 2B). Additionally, *bla*_NDM-1_ is also located on a truncated Tn*125*, which is captured by the integron In*1201* (Figure 3). Both two plasmids had conjugal transfer regions (Figure 2), and conjugation experiments also confirmed that these plasmids were capable of horizontal transfer between strains.

## Discussion

Carbapenem-resistant *K. oxytoca* complex, harboring various carbapenemase genes, is spreading globally (Hoenigl et al., 2012; Nazik et al., 2014; Wang et al., 2017), but molecular epidemiological studies are still limited. We conducted a multi-center study on clinical CRKO complex collected from five representative regions of China to probe its molecular epidemiological characteristics. In our study, *K. michiganensis* was the most common species, accounting for 64.0% of the total isolates, followed by *K. oxytoca* (20.0%, 5/25) and *K. pasteurii* (16.0%, 4/25). *K. grimontii*, *K. huaxiensis* and *K. spallanzanii* were not detected. *K. michiganensis* is phylogenetically closest to *K. oxytoca* (Saha et al., 2013). Previous studies have confirmed *K. michiganensis* as the most common species through analysis of 162 *K. oxytoca*-related whole-genome sequences (Chen et al., 2020), and suggested that some conditions attributed to *K. oxytoca* may actually be caused by *K. michiganensis*. Although current research on *K. michiganensis* is limited, it is considered an emerging pathogen closely related to human infections (Chen et al., 2020).

In this study, carbapenemase genes were detected in 92.0% (23/25) isolates, indicating that carbapenemase was also the most important carbapenem resistance mechanism in *K. oxytoca* complex. Unlike *K. pneumoniae*, of which KPC was the most prevalent carbapenemase in China (Zhang et al., 2017), Class B MBLs were more commonly detected in *K. oxytoca* complex in our study. Class B MBLs include NDM, IMP and VIM, with NDM being the most widespread MBLs in Enterobacteriaceae. In addition to the *K. oxytoca* complex, NDM was also frequently identified in *K. pneumoniae*, *E. coli*, *E. aerogenes*, *Serratia marcescens*, *Citrobacter freundii* and *E. cloacae* (Zhang et al., 2017). Among MBLs, the isolation rate of IMP and VIM in Enterobacteriaceae was usually lower than NDM. However, compared with Enterobacteriaceae of the same period, we found that the isolation rate of *bla*_IMP_ (26.9%) in *K. oxytoca* complex appears to be significantly higher. A nationwide surveillance of CRE strains in China also showed the similar result with the isolation rate of *bla*_IMP_ in *K. oxytoca* complex was 25%, much higher than other CRE strains during the same period (Zhang et al., 2017). However, all the *bla*_IMP_-harboring strains in this study were isolated in Suzhou, accounting for 50.0% (7/14) of the total strains from this site, among which 3 strains belonged to ST92, and the rest were of independent STs. Moreover, due to the long separation interval of the three ST92 strains (>6 months) and the fact that the patients were from different wards, the possibility of clonal transmission was low. Ceftazidime-avibactam, a novel β-lactam/β-lactamase inhibitor, is highly active against KPC-producing CRE, and has been the first-line therapy for CRE infections in many hospitals. Ceftazidime-avibactam susceptibility experiment was carried out on 7 strains only producing KPC-2, and the results showed that all strains were sensitive, indicating that ceftazidime-avibactam still maintained high efficacy to KPC-producing *K. oxytoca* complex *in vitro*. In this study, three strains co-harboring both class A and class B carbapenemase genes were also detected, which may bring more challenges to the clinical treatment as these strains may confer to high level carbapenem resistance and resistance to novel β-lactam/β-lactamase inhibitors. *K. ox*6089 and *K. ox*7525 were two non-carbapenemase-producing strains. Although *K. ox*6089 was found to harbor ESBLs *CTX-M-65*, we suspect additional mechanisms, such as mutations of outer membrane proteins or overexpression of efflux pumps, may contribute to carbapenem resistance in these strains.

Although *K. oxytoca* complex and *K. pneumonia* both belong to the genus of *Klebsiella*, the epidemic characteristics of the two are significantly different. The global spread of carbapenem resistant *K. pneumoniae* is mainly driven by a few high-risk clone group (CG) strains (Wyres and Holt, 2016). In China, the clonal spread of ST11 is the key cause of carbapenem resistance in *K. pneumoniae* (Qi et al., 2010; Zhang et al., 2017). On the contrary, our research revealed a polyclonal dissemination characteristic of *K. oxytoca* complex in China. No major STs were detected in *K. oxytoca* complex and the three species all showed the characteristics of polyclonal transmission. However, the distribution of STs is related to the species of *K. oxytoca* complex. Among them, ST43 and ST92 were mainly identified in *K. michiganensis*, while ST145 was a slightly common ST in *K. oxytoca*. Our study also showed that carbapenemase types may be related to the STs of *K. oxytoca* complex. As ST43 mainly produced KPC and ST92 mainly produced MBLs, especially IMP.

Different from *K. pneumoniae*, class B metallo-β lactamases (MBLs) were the most common carbapenemase in *K. oxytoca* complex, especially NDM-producing strains which have frequently been reported in hospitalized patients or hospital environment (Wang et al., 2017; Zhang et al., 2017; Pérez-Vazquez et al., 2019; Yang et al., 2022). In this study, two *bla*_NDM_-harboring plasmids were sequenced and characterized to be IncX3 and IncC incompatibility groups, respectively. In China, IncX3 is the most common type of *bla*_NDM_-harboring plasmid replicon in carbapenem-resistant Enterobacteriaceae (An et al., 2016; Wang et al., 2017). Conjugation experiments show that the IncX3 plasmid can be efficiently transferred to a variety of Enterobacteriaceae (Wang et al., 2018), which also makes the IncX3 plasmid an important vector for the global spread of *bla*_NDM_. In addition to the IncX3 plasmid, IncC plasmid carrying *bla*_NDM_ was detected in this study. IncC is another common type of *bla*_NDM_-carrying plasmid. It has a broad host range. Except for Enterobacteriaceae, it is also found in the *Morganellaceae* and *Vibrionaceae*, contributing to the global spread of *bla*_NDM_ (Wu et al., 2019). The IncC-type plasmid has been previously reported in *K. oxytoca* complex in Spain (Pérez-Vazquez et al., 2019). But in China, this was the first report about *bla*_NDM_-carrying IncC plasmid in *K. oxytoca* complex. In this study, conjugation experiments confirmed these plasmids had the ability of horizontal transfer between strains, which should be vigilant against the outbreaks of carbapenem-resistant bacteria caused by horizontal transmission of plasmids between strains.

The molecular epidemiological characteristics of CRKO complex in China appears to be significantly different from those in other countries. A study in Spain showed that ST2 dominated in *K. oxytoca* complex, while in China, ST43, ST92 and ST145 are relatively common, and the rest STs are distributed sporadically. Other studies also suggested limited international transmission of the same clone (Long et al., 2022), but close surveillance is needed to monitor the further spread of these multidrug resistant strains. In addition, the emergence of CP *K. oxytoca* in Spain was mainly due to the spread of isolates that produced VIM and OXA-48 (Pérez-Vazquez et al., 2019), while in China it was mainly NDM, KPC and IMP, and no isolates of VIM and OXA-48 were detected, revealing the complexity of the molecular resistance characteristics of *K. oxytoca* complex.

In summary, our study detected different carbapenemase types in various *K. oxytoca* complex STs from 5 regions in China, highlighting the extensive genetic diversity among *K. oxytoca* complex strains. Our study served as the first step towards detailing the genomic and clinical characterization of *K. oxytoca* complex strains in China. Given the close evolutionary relationship between *K. oxytoca* complex and *K. pneumoniae*, one of the most common MDR species, further comparison of the genomic and clinical characteristics between the two groups will likely help to unravel novel features associated with the epidemics of MDR *K. pneumoniae* and provide strategies to control the further spread of drug resistance.

## Data availability statement

The datasets presented in this study can be found in online repositories. The names of the repository/repositories and accession number(s) can be found in the article/Supplementary material.

## Author contributions

HY, MW, WJ, BH, FQ, and BS were responsible for the specimen collection and information collation. WW and XY were responsible for the experiment and article writing. Y-WT, LC and HD contributed to the revision of the article. All authors contributed to the article and approved the submitted version.

## Funding

This study was supported by the Science Foundation of Jiangsu Province Health Department [ZDB2020014]; the Science Foundation of Suzhou Health Department [LCZX202106] and the Discipline Construction Program of the Second Affiliated Hospital of Soochow University [XKTJ-TD202001]; the Science and Technology Program of Suzhou (SLJ2022003, 2022SS41).

## Conflict of interest

The authors declare that the research was conducted in the absence of any commercial or financial relationships that could be construed as a potential conflict of interest.

## Publisher’s note

All claims expressed in this article are solely those of the authors and do not necessarily represent those of their affiliated organizations, or those of the publisher, the editors and the reviewers. Any product that may be evaluated in this article, or claim that may be made by its manufacturer, is not guaranteed or endorsed by the publisher.

## Footnotes

^1^ https://pubmlst.org/

^2^ https://inkscape.org/en/

^3^ https://cge.food.dtu.dk/services/PlasmidFinder/

^4^ https://inkscape.org/
